# The Association Between Social Support and Suicidal Ideation Among Undergraduate Students: A Systematic Review and Meta-Analysis

**DOI:** 10.3390/ejihpe16050059

**Published:** 2026-04-23

**Authors:** Sijun Chen, Aqeel Khan, Mohd Rustam Mohd Rameli

**Affiliations:** Department of Educational Studies & Behavioral Sciences, Faculty of Educational Sciences & Technology (FEST), Universiti Teknologi Malaysia (UTM), Skudai 81310, Johor, Malaysia

**Keywords:** social support, suicidal ideation, undergraduate students, systematic review, meta-analysis, suicide prevention

## Abstract

**Background**: Suicide among emerging adults has become a significant global public health concern. Suicidal ideation is the prerequisite for suicide, and social support is recognized as a key protective factor against suicidal ideation. However, the relationship between the strength and consistency of social support and suicidal ideation among undergraduate students remains unclear. This study synthesized empirical studies to quantify the relationship between social support and suicidal ideation among undergraduate students and determine the different correlations between various sources of social support and suicidal ideation. **Methods**: A systematic review and meta-analysis was conducted following PRISMA 2020 guidelines. Five electronic databases (Web of Science, Scopus, PubMed, ProQuest, and ScienceDirect) were searched for studies published from 2016 to 2025. Eligible studies reported quantitative associations between social support and suicidal ideation among undergraduate students. Correlation coefficients were transformed using Fisher’s z and pooled using a random-effects model. Heterogeneity was evaluated using Cochran’s Q and I^2^ statistics. Risk of bias assessments, moderator analysis, sensitivity analysis, subgroup analysis, and publication bias assessments were conducted. **Results**: Fifteen studies with sixteen independent effect sizes and more than 26,000 participants were included. The meta-analysis showed a moderate negative association between social support and suicidal ideation (pooled r = −0.33, 95% CI [−0.40, −0.25]) under a random-effects model. A high heterogeneity was observed among studies (I^2^ = 97%, *p* < 0.001). There are no studies classified as having a high risk of bias. The standardized sample size demonstrated a significant moderating effect (β = 0.2568, *p* = 0.0022). Sensitivity analysis confirmed the stability of the pooled effect. Subgroup analysis indicated that the strength of the association between social support and suicidal ideation did not differ significantly between Asian and non-Asian studies. No significant publication bias was detected (Egger’s *p* = 0.19). Narrative synthesis further suggested that family support showed the most consistent protective association compared with friends’ support and support from others. **Conclusions**: Social support is moderately and consistently associated with reduced suicidal ideation among undergraduate students. These findings highlight social connectedness, particularly family support, as a central interpersonal protective factor and strengthen social support’s role in university suicide prevention initiatives.

## 1. Introduction

Suicide among emerging adults has become a significant global public health concern (World Health Organization, 2025). Contemporary theoretical frameworks, including the interpersonal–psychological theory of suicide (Joiner, 2005) and the ideation-to-action model (Klonsky & May, 2015), emphasize that the emergence of suicidal ideation and the progression to suicidal behaviors are governed by partially different mechanisms. Suicidal ideation represents a distinct stage in the suicidal process, rather than being interchangeable with broader outcomes such as suicide attempts or completed suicide. Undergraduates are at a higher risk because they often have to deal with academic pressure, developmental transitions, financial strain, and interpersonal challenges (Adams et al., 2016; March-Amengual et al., 2022; Mulaudzi, 2023). Suicidal ideation is the prerequisite for suicide and is relatively common among undergraduate students (Mortier et al., 2018; Sivertsen et al., 2025; Veloso & Lima, 2019). Recent global crises, particularly the COVID-19 pandemic, have further intensified psychological distress in this population, and numerous studies have shown that the rates of depression, anxiety and suicidal ideation have increased (Brenner & Bhugra, 2020; McLafferty et al., 2021; Nuñez Fadda et al., 2023; Zarowski et al., 2024), thereby underscoring the urgent need to identify modifiable risk and protective factors within higher-education settings (Cecchin & Murta, 2025).

Social support, defined as the perceived or actual availability of emotional, informational, and instrumental assistance from one’s social network, is widely recognized as a key protective factor for mental health (S. Cohen & Wills, 1985; Martínez-Rives et al., 2025; Parker et al., 2021; Thoits, 2011). The interpersonal–psychological theory of suicide posits that thwarted belongingness is a proximal contributor to suicidal ideation (Joiner, 2005), suggesting that supportive social connections may reduce suicide risk. Stress-buffering models of social support propose that social support can mitigate the stressors’ negative psychological impact (S. Cohen & Wills, 1985). Consistent with these theoretical perspectives, empirical studies have demonstrated significant negative associations between social support and suicidal ideation among undergraduate students across diverse cultural contexts (Chu et al., 2021; Hollingsworth et al., 2018; Howlader et al., 2024; Hussein & Yousef, 2024). 

Despite the growing number of relevant studies, there are still gaps in this field. Although longitudinal designs are better suited to clarify temporal and causal relationships between social support and suicidal ideation, the current body of literature focusing on undergraduate populations is predominantly based on cross-sectional studies. In this review, no eligible longitudinal studies met the inclusion criteria. This reflects a limitation in the existing evidence base rather than a restriction imposed by the study design of this review. The results of the existing work vary in terms of the strength of the association between social support and suicidal ideation, which is partly due to differences in research design, measurement methods, and social–cultural background. Although previous reviews have examined the relationship between social support and suicidal ideation among adolescents and older adults (Miller et al., 2015; Wan et al., 2026), few have specifically focused on undergraduate students. Also, these reviews do not distinguish between different sources of social support and suicidal ideation, such as family, friends, and others. There has been evidence suggesting that these sources may result in differential protective effects. It is particularly important to fill this gap upon the realization that undergraduate students face unique intellectual and interpersonal relationship issues, which may influence the dynamics of social support in relation to suicidal ideation. 

In this study, we mainly address the following research questions: (1) What is the strength of the association between social support and suicidal ideation among undergraduate students? (2) Which sources of social support are most consistently associated with reduced suicidal ideation? This review aims to advance the understanding of how social support relates to suicidal ideation among undergraduate students and to guide future research on suicide intervention development in higher education.

## 2. Methods

This review and meta-analysis was conducted and reported following the PRISMA 2020 guidelines. The review protocol was not prospectively registered in PROSPERO because our study focused on psychosocial observational evidence rather than clinical intervention outcomes. All methodological procedures were predefined prior to data extraction to ensure transparency and reproducibility.

### 2.1. Search Strategy

A systematic literature search was conducted in Web of Science Core Collection, Scopus, PubMed, ProQuest, and ScienceDirect for English-language publications from January 2016 to December 2025. A combination of free-text terms and database-specific controlled vocabulary was used, including (“social support” OR “perceived social support”) AND (“suicidal ideation” OR “suicidal thought”) AND (“undergraduate students” OR “undergraduate students” OR “university students” OR “college students”). To maintain conceptual clarity and ensure the comparability of effect sizes, the search strategy was intentionally restricted to studies examining suicidal ideation, excluding broader suicide-related outcomes such as suicide attempts or suicidal behaviors. This decision is supported by theoretical frameworks, including the interpersonal–psychological theory of suicide (Joiner, 2005) and the ideation-to-action model (Klonsky & May, 2015), which conceptualize suicidal ideation as a distinct stage with different underlying mechanisms from suicidal behaviors.

### 2.2. Eligibility Criteria

Empirical studies that reported quantitative group statistics were included if they met the following PICOS criteria (Table 1): (a) Population: undergraduate students of any age, gender, or nationality; (b) Exposure: social support, measured by a validated scale or direct self-report items; (c) Outcome: suicidal ideation, measured by a validated scale or direct self-report items; (d) Study Design: quantitative studies reporting the correlation coefficient (r) between overall social support and suicidal ideation. No restrictions were imposed regarding study design, such as cross-sectional or longitudinal.

Studies were excluded if they: (a) did not focus on undergraduate students; (b) did not report extractable data on social support (overall); (c) did not report extractable data on suicidal ideation; or (d) were qualitative studies, reviews, case reports, or non-empirical publications.

### 2.3. Study Selection

**Figure 1:**
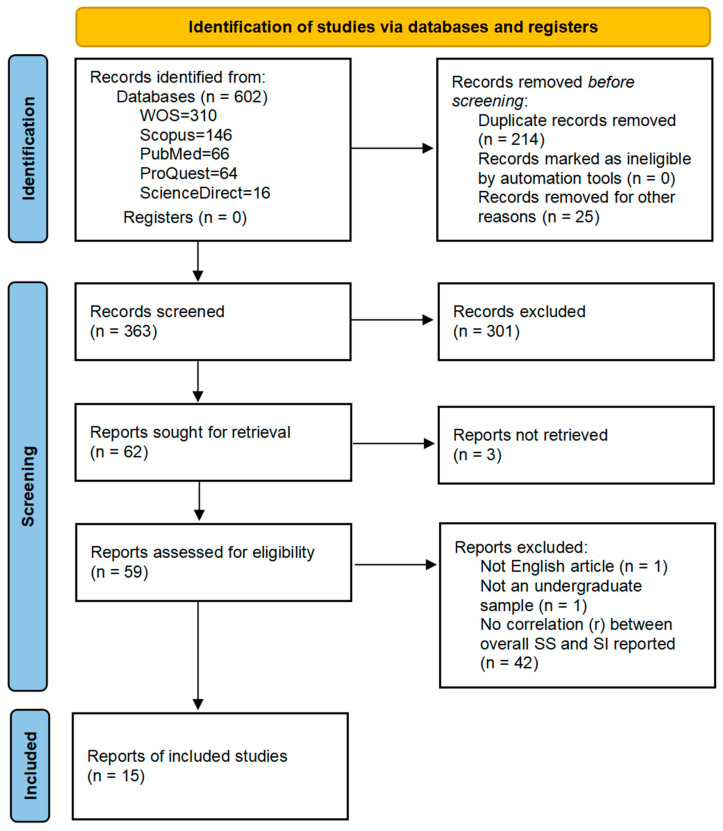
PRISMA flow diagram.

Search results were imported into reference management software (Zotero 7.0), and duplicates and non-articles were removed. Titles and abstracts were independently screened by two reviewers (CSJ and MRMR) against the eligibility criteria. The full texts of potentially relevant articles were then assessed. Any disagreements were resolved through discussion or consultation with a third reviewer (AK). The PRISMA flowchart presented in Figure 1 illustrates this review process.

### 2.4. Data Extraction

A standardized data extraction form was used to collect information from each included study: author(s) (year), country, study design, sample, sample size, female (%), age (SD), social support measurement, suicidal ideation measurement, and Pearson’s correlation (r) between overall social support and suicidal ideation (Table 1).

### 2.5. Risk of Bias Assessment

The methodological quality of the included studies was assessed using the Joanna Briggs Institute (JBI) Analytical Cross-Sectional Checklist, as all fifteen eligible studies employed cross-sectional observational designs. Two authors (CSJ and MRMR) independently evaluated the methodological risk of bias of the included studies, and any disagreements were resolved through discussions with AK. Each study was rated as Yes, No, or Unclear for each criterion, and an overall risk of bias judgment was determined following JBI guidance. Following previous systematic review conventions, studies meeting seven to eight criteria were classified as low risk of bias, those meeting five to six criteria as moderate risk, and studies meeting four or fewer criteria as high risk of bias.

### 2.6. Statistical Analysis

In this study, we used Review Manager (RevMan) version 5.4 to conduct analyses. This study uses correlation coefficients r as the primary effect size indicator, but the standard error of Pearson’s correlation coefficient varies with the magnitude of r. This characteristic makes the direct pooling of correlation coefficients inappropriate in meta-analytic procedures, so we transformed all correlation coefficients into Fisher’s z values before conducting statistical synthesis. This transformation stabilizes variance and improves the approximation of a normal sampling distribution. After the meta-analysis, we converted the pooled Fisher’s z estimates back to correlation coefficients for better interpretation. In addition, we interpreted the effect sizes according to J. Cohen (1992) guidelines, where r = 0.10 indicates a small effect, r = 0.30 indicates a moderate effect, and r = 0.50 indicates a large effect.

We adopted a random-effects model to account for expected between-study variability (Hedges & Vevea, 1998). We use Cochran’s Q statistic and the I^2^ statistic to assess statistical heterogeneity. I^2^ values of 25%, 50%, and 75% were interpreted as low, moderate, and high heterogeneity respectively (Higgins et al., 2003). Under the random-effects model, study weights were calculated based on both within-study variance and between-study variance (τ^2^). Given the high heterogeneity observed, τ^2^ contributed substantially to the weighting scheme, reducing the relative influence of sample size on study weights. We evaluated publication bias using funnel plots to visually inspect the symmetry of effect size distributions. The analysis also applied Egger’s regression test to statistically assess funnel plot asymmetry (Egger et al., 1997).

## 3. Results

### 3.1. Study Selection

The initial database search identified 602 records. After removing 214 duplicates and 25 non-article records, 363 records remained for title and abstract screening, leaving 62 articles. Since three records were not retrieved, 59 articles were deemed potentially eligible for full-text review. Of these, 44 full-text articles were excluded due to reasons such as being a non-English article, having an inappropriate study population, or having insufficient statistical data. Ultimately, 15 studies met the inclusion criteria and were included in the final systematic review. The study selection process is illustrated in the PRISMA flow diagram (Figure 1).

### 3.2. Study Characteristics

The fifteen included studies, published between 2016 and 2025, examined the association between social support and suicidal ideation among undergraduate students. All studies employed a cross-sectional observational design and were conducted in university-based samples.

One cross-national study (Siegmann et al., 2018) was conducted in Germany and China. The fifteen included studies contributed 16 independent samples. The studies were conducted across multiple countries, with a clear predominance of research conducted in Asia (68.8%). Most Asian studies were conducted in China (*n* = 7), followed by Malaysia (*n* = 2), India (*n* = 1) and the Philippines (*n* = 1). Outside Asia, two studies were conducted in the USA, and one study each was conducted in Germany, Egypt, and Colombia. The predominance of studies conducted in Asian countries may introduce potential source bias and limit the generalizability of the findings to non-Asian populations.

Sample sizes ranged from 200 to 9716 undergraduate students, and the total pooled sample size exceeded 26,000 participants. Most studies included both male and female students, although one study did not report detailed gender distributions. Where reported, participants were predominantly young adults, with mean ages generally falling within the late adolescence to early adulthood range, consistent with typical undergraduate populations. 

Social support was measured using a number of standardized self-report instruments. The most used one that is present in seven studies is the Multidimensional Scale of Perceived Social Support (MSPSS). Two studies involved the use of the Perceived Social Support Scale (PSSS). Other instruments were each used in one study, including the Oslo Social Support Scale (OSSS-3), the Social Support Rating Scale (SSRS), the Inventory of Socially Supportive Behaviors (ISSB), the F-SozU, the Social Support Appraisals Scale (SSAS), and the Scale of Perceived Social Support (SPSS).

Suicidal ideation was assessed using several validated self-report measures. The Suicidal Behaviors Questionnaire—Revised (SBQ-R) was the most commonly used instrument, appearing in five studies. The Self-Rating Idea of Suicide Scale (SIOSS) was used in two studies. Other measures, each used in a single study, included the HDSQ-SS, the Suicidal Ideation Scale (SIS), a single-item measure from the revised Symptom Checklist (SCL-90-R), the Chinese version of the Beck Scale for Suicidal Ideation (BSSI-CV), the Scale for Suicide Ideation (SSI), the Adult Suicide Ideation Questionnaire (ASIQ), BDI Item 9 and items derived from the National Comorbidity Survey (NCS-SSI).

All included studies reported Pearson’s correlation coefficients (r) describing the association between social support and suicidal ideation, allowing for direct inclusion in the meta-analysis. Detailed characteristics of the included studies are presented in Table 2. Across studies, higher levels of perceived social support were consistently associated with lower levels of suicidal ideation, although the magnitude of the associations varied across studies and measurement approaches.

### 3.3. Risk of Bias

Two authors (CSJ and MRMR) independently evaluated the methodological risk of bias of the included studies by using the Joanna Briggs Institute (JBI) Analytical Cross-Sectional Checklist, and any disagreements were resolved through discussions with AK. A summary of the assessments with detailed item-level ratings is provided in Table 2. Twelve studies were rated to be at a low risk of bias, while three studies had a moderate risk of bias. This may be due to insufficient reporting of confounding control or measurement validity. There are no studies classified as having a high risk of bias. These findings suggest that the methods used in this study are generally acceptable, although there might be limitations inherent to cross-sectional designs.

### 3.4. Overall Meta-Analysis

In this study, fifteen studies contributed sixteen independent effect sizes because there is one cross-national study (Siegmann et al., 2018) that reported separate correlation coefficients for two independent samples from two different countries. As these samples were recruited independently and analyzed separately, each effect size was treated as an independent observation in the meta-analysis, following established methodological recommendations.

**Figure 2:**
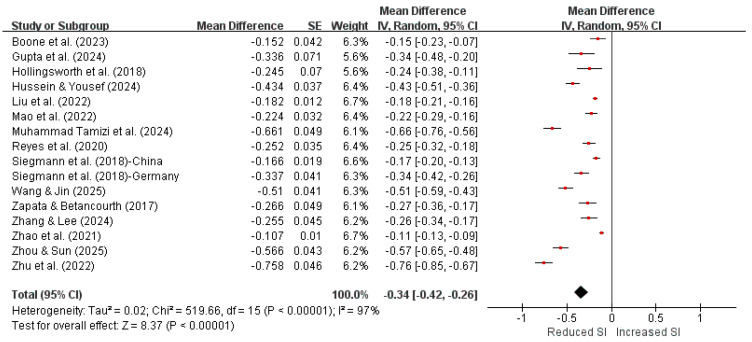
Forest plot (Fisher’s z transformed).

The meta-analysis showed a moderate negative association between perceived social support and suicidal ideation (Fisher’s z = −0.34, 95% CI: [−0.42, −0.26]). Pearson’s r value (r = −0.33, 95% CI [−0.40, −0.25]) suggested that higher levels of perceived social support were associated with lower levels of suicidal ideation among undergraduate students. Substantial heterogeneity was observed across studies (Q = 519.66, df = 15, *p* < 0.001; I^2^ = 97%), indicating considerable variability in effect sizes beyond sampling error. The individual study effect sizes and the pooled estimate are presented in Figure 2.

### 3.5. Moderator Analysis

Meta-regression analyses were conducted to explore whether study-level characteristics could explain the observed between-study heterogeneity in effect sizes. The results showed that average age was not a significant moderator (β = 0.0346, *p* = 0.2141), indicating that differences in participants’ age did not contribute to the heterogeneity. Similarly, gender composition was not significantly associated with effect size (β = 0.6994, *p* = 0.2080), suggesting that the proportion of female participants did not account for variability across studies. In addition, the year of publication was not a significant moderator (β = −0.0329, *p* = 0.1090), indicating that the strength of the association has remained relatively stable over time. 

**Figure 3:**
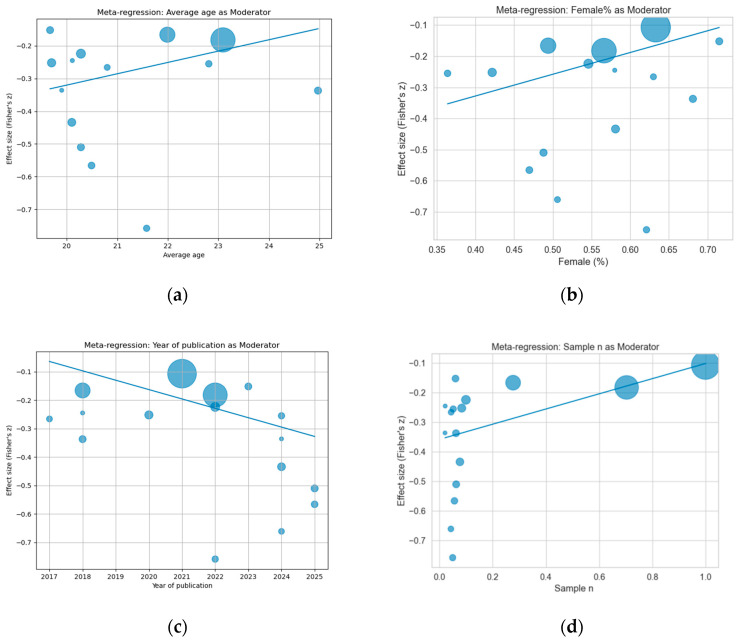
Meta-regression. (**a**) Average age, (**b**) gender composition, (**c**) year of publication, and (**d**) sample size.

In contrast, the standardized sample size demonstrated a significant moderating effect (β = 0.2568, *p* = 0.0022), as seen in Figure 3. Given that the overall pooled effect size was negative, this positive coefficient indicates that studies with larger sample sizes tended to report effect sizes closer to zero, reflecting a weaker association. Conversely, smaller studies tended to report stronger (more negative) effect sizes. This pattern suggests a potential small-study effect and indicates that sample size may partially explain the observed heterogeneity, or possibly reflects differences in statistical power and study precision.

However, since only one moderator was significant while the others were not, a substantial proportion of heterogeneity likely remains unexplained, implying that additional unmeasured factors (e.g., measurement tools, study design, or cultural context) may also contribute to the variability in effect sizes across studies.

### 3.6. Sensitivity Analysis

We use leave-one-out sensitivity analysis to evaluate the influence of individual studies on the overall association between social support and suicidal ideation. Each study was sequentially removed, and the pooled effect size was recalculated using a random-effects model. The results indicated that the pooled correlation coefficients ranged from approximately r = −0.30 to r = −0.35. The direction and magnitude of the association remained consistent across all analyses. No single study exerted a disproportionate influence on the overall effect size, suggesting that the findings were robust.

### 3.7. Subgroup Analysis

Subgroup analyses were conducted based on geographic region (Asian and non-Asian) to explore potential cultural differences in the association between social support and suicidal ideation. This classification was theoretically informed by prior cross-cultural research suggesting that collectivist versus individualist cultural orientations may influence both the perception of social support and the expression of suicidal ideation. Previous cross-cultural research has suggested that these factors may vary substantially between Asian and Western contexts (Eskin et al., 2020). 

**Figure 4:**
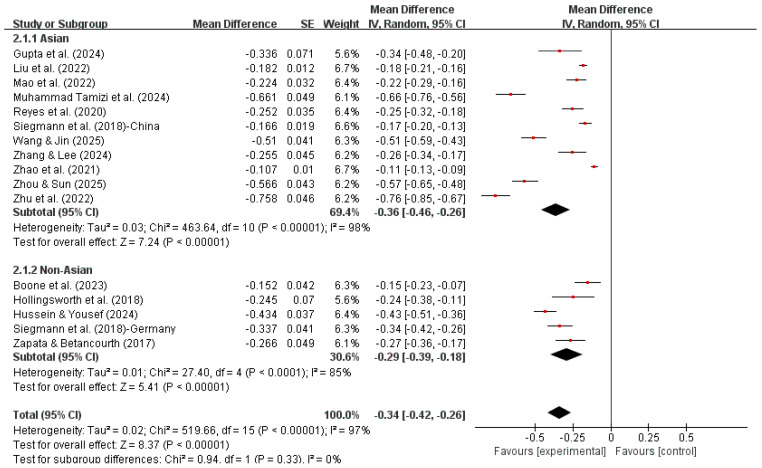
Subgroup analysis (Asian studies and non-Asian studies).

In Asian studies, social support showed a moderate negative association with suicidal ideation (r = −0.35, 95% CI [−0.43, −0.25]). In non-Asian studies, the association remained statistically significant but was smaller in magnitude (r = −0.28, 95% CI [−0.37, −0.18]). The test for subgroup differences was not statistically significant (χ^2^ = 0.94, df = 1, *p* = 0.33), indicating that the strength of the association between social support and suicidal ideation did not differ significantly between Asian and non-Asian studies, which suggests that the overall findings are not solely driven by Asian studies. However, high levels of heterogeneity were still observed within both subgroups (Asian: I^2^ = 98%; non-Asian: I^2^ = 85%), suggesting that it alone is insufficient to explain heterogeneity (Figure 4).

### 3.8. Publication Bias

**Figure 5:**
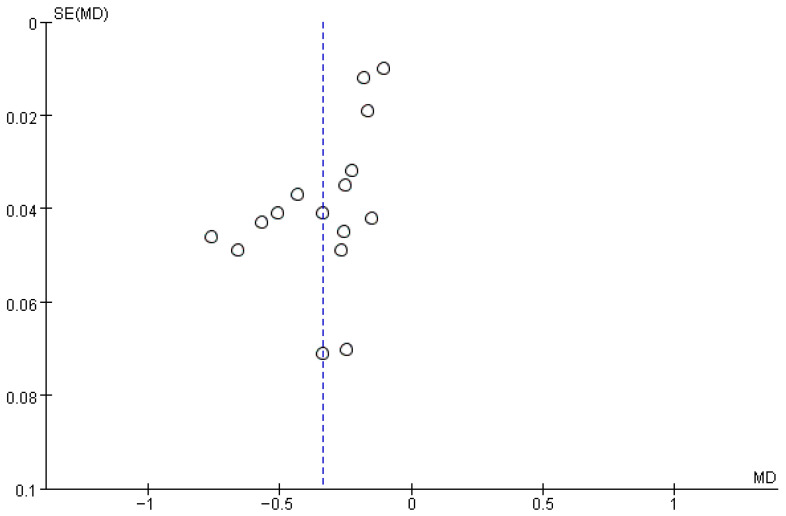
Funnel plot.

To assess potential publication bias, a funnel plot was generated by plotting effect sizes against their standard errors. The funnel plot in Figure 5 suggested minor asymmetry. However, Egger’s regression test did not indicate significant publication bias, with *p* = 0.19. The findings demonstrate a robust negative association between social support and suicidal ideation among undergraduate students, although the high level of heterogeneity suggests that study characteristics such as measurement instruments, cultural context, and sample composition may contribute to the observed variability. These findings suggest that the observed association is unlikely to be substantially influenced by small-study effects. Given the substantial heterogeneity across studies, the results of publication bias analyses still should be interpreted with caution.

### 3.9. Comparative Effects of Different Sources of Social Support on Suicidal Ideation

As we can see from Table 3, there are eight studies giving effect sizes between the different sources of social support and suicidal ideation. A key finding is that the protective association between social support and suicidal ideation among undergraduate students is not consistent across sources of support. Due to study limitations, a quantitative meta-analysis of source-specific support was not feasible; therefore, a narrative synthesis was conducted.

When comparing the relative contributions of different sources of perceived social support, we observed a consistent hierarchical pattern across different studies. Family support demonstrated the most stable and robust protective association with suicidal ideation. The majority of studies reported significant negative relationships between family support and suicidal ideation, although different analytical approaches, e.g., structural equation modeling, regression analyses, and logistic regression models, are used. Family support often remained significant after adjustment for psychological covariates, suggesting an independent protective effect.

Evidence regarding friends and others’ support was less consistent. Few studies reported significant protective associations, and several studies either found no significant effects or did not examine them. Although several studies identified significant negative bivariate associations, the effect of friends and others’ support frequently became insignificant in multivariable models controlling for depressive symptoms, stress, or related psychosocial factors. This means that the protective role of friends and others’ support may operate indirectly through emotional or psychological mediators rather than exerting a strong independent effect. The variability in operational definitions and measurement approaches across studies may also partially explain these inconsistencies. 

We find that family support represents the most consistently supported protective factor against suicidal ideation among undergraduate students, followed by weaker and less stable effects for friends and others’ support. However, these results should be interpreted cautiously because effect sizes were not directly comparable across analytical approaches.

## 4. Discussion

### 4.1. Summary of Main Findings

This review synthesized empirical studies published between 2016 and 2025 to examine the strength of the relationship between perceived social support and suicidal ideation among undergraduate students. The pooled analysis showed a moderate negative relationship between perceived social support and suicidal ideation (r = −0.33). In general, students who reported higher perceived social support also reported fewer suicidal thoughts. This pattern appeared across studies from different countries and universities. This association was further supported by sensitivity analyses. The pooled effect size remained relatively stable when the individual studies were removed one after another, in the course of the analysis. This finding shows that the general relationship was not necessarily represented by some specific study but was rather a common trend in the available literature. 

We had similar observations in the subgroup analyses of Asian and non-Asian samples. Even though the effect of correlation was seen to be perhaps a bit stronger in Asian samples, the inter-regional difference was not statistically significant. Such results indicate that the relationships between perceived social support and suicidal ideation occur in different cultural settings. Previous cross-cultural studies have also proposed that the existence of supportive interpersonal relationships is one of the key protective elements against suicidal risk among young adults (Martínez-Rives et al., 2025). We also conducted moderator analysis to explore potential moderators of this association. Meta-regression results indicate that factors like average age, gender composition, and the year of publication did not significantly influence the effect size, but sample size acted as a significant moderator. 

Several studies in this review reported that family support was still linked to lower suicidal ideation even after controlling for psychological factors such as depression. Previous research has also shown that family support can weaken the impact of psychological distress on suicidal ideation and improve psychological adjustment among university students (Blessing et al., 2025; Wei et al., 2024). Supportive friendships can provide emotional comfort and companionship, but evidence about friends and others’ support is less consistent (Dong & Zhao, 2023; Ogba et al., 2025). Some studies suggest that the relationship between friends’ support and suicidal ideation may depend on other psychological factors such as perceived burdensomeness or depressive symptoms (Hollingsworth et al., 2018; Khan et al., 2023). These findings suggest that friends and others’ support may affect suicidal ideation in an indirect way instead of acting as a strong independent protective factor.

### 4.2. Sources of Heterogeneity

This meta-analysis found a high heterogeneity across studies (I^2^ = 97%), and this suggests that the differences in effect sizes are larger than what would be expected from sampling error. Such a high heterogeneity may exist in meta-analyses that combine psychosocial observational studies from different populations and research settings (Borenstein et al., 2009; Higgins et al., 2003).

High heterogeneity can be caused by cultural and contextual factors. A substantial proportion of studies were conducted in Asian countries, particularly China, which may represent a significant potential source of heterogeneity and limit the generalizability of the findings in this review. Although subgroup analyses comparing Asian and non-Asian samples did not reveal statistically significant differences, we should not consider this as evidence of cultural equivalence. Instead, it may be due to limited statistical power, measurement variability, or the broad categorization of cultural contexts.

From a theoretical perspective, cultural norms may influence how social support is perceived and utilized. In collectivist contexts, which are quite common in many Asian societies, family relationships are often characterized by interdependence and relational obligations. This enhances the protective role of family support by strengthening belongingness and reducing interpersonal vulnerability. On the other hand, in more individualistic contexts, social support may be more peer-oriented and operate through different psychological pathways, such as autonomy support. Also, cultural differences in stigma, emotional expression, and help seeking behaviors may influence how suicidal ideation is reported and may potentially contribute to the variability in effect sizes across studies.

Meta-regression analyses further explored potential sources of heterogeneity. The results indicated that average age, gender composition and year of publication were not significant moderators, but sample size showed a significant moderating effect. Specifically, larger studies tended to report effect sizes closer to zero, while smaller studies reported stronger associations. This pattern suggests the presence of a small study effect and indicates that study precision may partially contribute to the observed heterogeneity.

The relatively similar weighting of studies reflects the use of a random-effects model, where weights are determined by both within-study variance and between-study variance (τ^2^). The very high heterogeneity (I^2^ = 97%) indicates substantial between-study variability, suggesting that true effect sizes differ across studies. As τ^2^ increases, it contributes more to the total variance, diminishing the influence of within-study variance driven by sample size. As a result, larger studies do not receive disproportionately higher weights, which leads to a more balanced distribution.

Different measurements of perceived social support and suicidal ideation were used. This difference can be attributed to a number of factors. In the first case, the social support instruments in the included studies included MSPSS, PSSS, SSRS, OSSS-3, and so on, which have varied definitions and outlines. The difference can alter the way social support is measured in different studies. In addition, the suicidal ideation instruments are the SBQ-R, the SIOSS, the SSI, and so on. Such instruments vary in terms of recall time and symptom coverage. The differences can lead to different effect sizes.

Despite this heterogeneity, a number of the results demonstrate that the general relationship is consistent. Sensitivity analyses revealed that the pooled effect size remained stable when we removed individual studies. Subgroup analyses also showed negative relationships across regions. These results suggest that the strength of the relationship may be different depending on the contexts, but the direction of the relationship remains similar.

### 4.3. Theoretical Implications

Some theoretical perspectives can help understand the association presented between perceived social support and suicidal ideation. One of the most frequently cited is the interpersonal–psychological theory of suicide proposed by Joiner (2005). This theory suggests that when an individual is in two different interpersonal states simultaneously, they are more likely to have suicidal thoughts, namely a sense of belonging frustration and a perception of being a burden. Sense of belonging frustration refers to an individual's long-term feeling of lacking a connection with others and believing that they do not have stable and meaningful interpersonal relationships. Perceiving being a burden is expressed as a belief that one’s existence brings pressure or distress to others. Perceiving social support may alleviate the interpersonal vulnerabilities of these individuals by enhancing their sense of social connection. Supportive relationships often provide emotional understanding and affirmation, as well as a sense of closeness among people, and make individuals feel valuable within their social network. Undergraduate students who can perceive stable support from family members, friends, or other important people usually develop a stronger sense of belonging. Stronger interpersonal connections help reduce social isolation. Reductions in social isolation may further decrease the risk of individuals having suicidal thoughts. Previous studies have reported that stronger perceptions of social connectedness are associated with lower suicidal ideation among university students (Hussein & Yousef, 2024).

The social support stress-buffering model proposed by S. Cohen and Wills (1985) also provides another explanation for this research result. Undergraduate students often need to deal with multiple sources of stress simultaneously. The requirements of academic tasks, the developmental transitions in early adulthood, and the economic pressure related to higher education can all become continuous stressful situations. Supportive social relationships may influence students’ perception of these pressures. Supportive relationships can assist people to overcome this set of challenges as they can offer emotional support, practical assistance, and information guidance. It can be said that such resources have the potential to alleviate the psychological effect of stressful events and, therefore, decrease the risk of developing suicidal ideation (Thoits, 2011).

### 4.4. Limitations and Future Direction

Several limitations should be considered when interpreting the findings of this study. Firstly, one of the main limitations of this study is that the included studies were all based on cross-sectional designs. Although this reflects the current state of the literature rather than a deliberate exclusion in methodology, cross-sectional designs limit conclusions about temporal ordering and therefore do not allow strong causal inferences regarding the relationship between perceived social support and suicidal ideation. Secondly, this study focused specifically on suicidal ideation rather than broader suicide-related outcomes. While this approach enhances conceptual precision, it also reflects theoretical distinctions emphasized in contemporary models of suicidal behavior, which differentiate the emergence of suicidal ideation from the progression to suicidal action, but potential articles may be missed. Another important limitation relates to the potential source bias arising from the geographic distribution of the included studies. Cultural differences in social norms, family structures, stigma, and help seeking behaviors may influence both perceived social support and the reporting of suicidal ideation. The majority of the included research that is being carried out in Asian countries might also limit the generalizability of the findings to more culturally diverse student populations. Although subgroup analyses suggested that the association remains consistent across regions, the relatively small number of non-Asian studies reduces the overall confidence. Fourth, the heterogeneity in the included studies was significant, meaning that the strength of the association might differ in different research settings. Statistical tests suggested that publication bias was not substantial; however, given the high heterogeneity, the findings should be interpreted with caution.

Future research should use longitudinal designs, experimental approaches, or mixed-methods research designs to clarify the temporal and causal mechanisms underlying the relationship between social support and suicidal ideation. Expanding research to more culturally diverse populations and employing standardized measurement tools or incorporating multi-method assessment approaches (such as combining self-report, behavioral, and clinical measures) would further improve the generalizability of the findings. Future research may also extend this work by examining whether the role of social support differs across stages of the suicidal process, which will contribute to the understanding of how social support operates across different stages of the suicidal process. In addition, future studies should also examine potential mediating and moderating factors, such as resilience, depressive symptoms, and perceived burden.

## 5. Conclusions

This review examined the strength of the association between social support and suicidal ideation among undergraduate students. The pooled results indicated a moderate negative relationship, which suggested that students who perceive higher levels of social support tend to report lower levels of suicidal ideation. This pattern appeared relatively consistent across the included studies despite differences in cultural contexts and measurement approaches. Moderator analyses suggested that sample size contributed to this variability, with smaller studies reporting stronger associations. The study also indicated that different sources of social support may contribute differently to this relationship. Evidence across studies suggested that family support demonstrated the most stable protective association, whereas support from friends and other significant individuals showed less consistent effects. These findings highlight the importance of interpersonal resources in understanding suicidal ideation among undergraduate students and suggest that strengthening supportive social environments may represent an important component of suicide prevention efforts in higher education. Future research should focus on longitudinal and experimental designs, culturally diverse populations, and standardized measurement approaches to better understand the mechanisms underlying this association.

## Figures and Tables

**Table 1:** Data extraction of the included studies.

No.	Study	Country	Study Design	Sample	Sample Size	Female (%)	Age, Mean Years (SD)	Instrument for SI	Instrument for SS	Pearson’s Correlation (r)
1	Boone et al. (2023)	United States	CS	Undergraduate students	583	417 (71.5%)	19.67 (4.16)	SBQ-R	MSPSS	−0.151
2	Gupta et al. (2024)	India	CS	University students	200	Not provided	19.9 (1.43)	SBQ-R	MSPSS	−0.323
3	Hollingsworth et al. (2018)	United States	CS	College students	207	120 (58%)	20.11 (SD not provided)	HDSQ-SS	MSPSS	−0.24
4	Hussein and Yousef (2024)	Egypt	CS	University students	745	433 (58.1%)	20.1 (1.4)	SIS	OSSS-3	−0.409
5	Liu et al. (2022)	China	CS	College students	6834	3818, (56.61%)	23.09 (4.82)	SCL-90-R	MSPSS	−0.18
6	Mao et al. (2022)	China	CS	Undergraduate students	964	526 (54.6%)	20.28 (1.79)	BSSI-CV	SSRS	−0.22
7	Tamizi et al. (2024)	Malaysia	CS	University students	415	210 (50.6%)	Not provided	SSI	MSPSS	−0.579
8	Reyes et al. (2020)	Philippines	CS	Colleges and universities students	811	342 (42.17%)	19.70 (1.49)	ASIQ	ISSB	−0.247
9	Siegmann et al. (2018)	Germany China	CS	University students	Germany: 601; China: 2687	Germany: 409 (68.1%); China: 1327 (49.4%)	Germany: 24.97 (4.84) China: 21.99 (1.19)	SBQ-R	F-SozU	Germany: −0.325 China: −0.164
10	Wang and Jin (2025)	China	CS	College students	608	297 (48.8%)	20.28 (2.05)	SIOSS	PSSS	−0.47
11	Zapata Roblyer and Betancourth Zambrano (2017)	Colombia	CS	College students	424	267 (63%)	20.8 (2.5)	SBQ-R	MSPSS	−0.26
12	Zhang and Lee (2024)	Malaysia	CS	College students	500	182 (36.40%)	22.81 (2.57)	BDI	SSAS	−0.25
13	Zhao et al. (2021)	China	Repeated CS	Undergraduate students	9716	6148 (63.3%)	17–24 years	NCS-SSI	MSPSS	−0.107
14	Zhou and Sun (2025)	China	CS	Undergraduate students	545	256 (46.97%)	20.49 (1.59)	SIOSS	PSSS	−0.512
15	Zhu et al. (2022)	China	CS	College students	480	298 (62.1%)	21.58 (1.98)	SBQ-R	SPSS	−0.64

Note: CS = Cross-sectional design.

**Table 2:** Risk of bias assessment of included studies.

No.	Study	Design	Q1	Q2	Q3	Q4	Q5	Q6	Q7	Q8	Yes (Total)	Risk of Bias
1	Boone et al. (2023)	CS	Yes	Yes	Yes	Yes	Yes	Unclear	Yes	Yes	7	Low
2	Gupta et al. (2024)	CS	Yes	Yes	Yes	Yes	Yes	Unclear	Yes	Yes	7	Low
3	Hollingsworth et al. (2018)	CS	Yes	Yes	Yes	Yes	Unclear	Unclear	Yes	Yes	6	Moderate
4	Hussein and Yousef (2024)	CS	Yes	Yes	Yes	Yes	Yes	Unclear	Yes	Yes	7	Low
5	Liu et al. (2022)	CS	Yes	Yes	Yes	Yes	Yes	Yes	Yes	Yes	8	Low
6	Mao et al. (2022)	CS	Yes	Yes	Yes	Yes	Yes	Yes	Yes	Yes	8	Low
7	Tamizi et al. (2024)	CS	Yes	Yes	Yes	Yes	Yes	Yes	Yes	Yes	8	Low
8	Reyes et al. (2020)	CS	Yes	Yes	Yes	Yes	Unclear	Unclear	Yes	Yes	6	Moderate
9	Siegmann et al. (2018)	CS	Yes	Yes	Yes	Yes	Unclear	Unclear	Yes	Yes	6	Moderate
10	Wang and Jin (2025)	CS	Yes	Yes	Yes	Yes	Yes	Yes	Yes	Yes	8	Low
11	Zapata Roblyer and Betancourth Zambrano (2017)	CS	Yes	Yes	Yes	Yes	Yes	Yes	Yes	Yes	8	Low
12	Zhang and Lee (2024)	CS	Yes	Yes	Yes	Yes	Yes	Unclear	Yes	Yes	7	Low
13	Zhao et al. (2021)	CSl	Yes	Yes	Yes	Yes	Yes	Yes	Yes	Yes	8	Low
14	Zhou and Sun (2025)	CS	Yes	Yes	Yes	Yes	Yes	Yes	Yes	Yes	8	Low
15	Zhu et al. (2022)	CS	Yes	Yes	Yes	Yes	Yes	Yes	Yes	Yes	8	Low

Note: CS = cross-sectional.

**Table 3:** Different sources of social support and suicidal ideation.

No.	Study	Country	Study Design	Sample	Sample Size	Female %	Age Mean (SD)	Instrument for SI	Instrument for SS	Method	Effect Size SS → SI (Family)	Effect Size SS → SI (Friends)	Effect Size SS → SI (Others)
1	Blessing et al. (2025)	USA	CS	Undergraduate students	928	667, (71.9%)	20.30 (4.31)	DSI-SS	MSPSS	Hierarchical regression	b = −0.04, *p* < 0.01 (significant)	b = −0.02, *p* = 0.417 (not significant)	b = −0.01, *p* = 0.301 (not significant)
2	Dong and Zhao (2023)	China	CS	Undergraduate students	469	349 (74.4%)	20 (1.18)	BSI-CV	MSPSS	SEM	β = −0.111 *p* < 0.05 (significant)	β = −0.038, *p* = 0.403 (not significant)	β = −0.051, *p* = 0.243 (not significant)
3	Duygulu et al. (2023)	Turkey	CS	Undergraduate students	1920	1373, 71.5%	21.26 (3.5)	Adapted questionnaire (based on ECOM)	Adapted questionnaire (based on ECOM)	Logistic regression	OR = 2.711 *p* < 0.001 (significant)	OR = 1.432 *p* = 0.013 (significant)	OR = 1.562 *p* < 0.001 (significant)
4	Lee et al. (2024)	China	CS	University/ college students	1359	882 (64.9%)	Not reported	PHQ-9	Single-item	Network analysis	r = −0.09 (negative partial correlation) (significant)	r = −0.05 (negative partial correlation) (significant)	Not examined
5	Macalli et al. (2018)	France	CS	University students	10,015	7579 (75.7%)	20.0 (1.8)	Single-item self-report	Single-item self-report	Logistic Regression	aOR = 8.58 (significant)	Not examined	Not examined
6	Miranda-Mendizabal et al. (2019)	Spain	CS	University students	2105	1166 (55.4%)	Range: 18–24	SITBI and C-SSRS	Adapted items from CIDI 3.0, PSSM, ACE, CTQ	Logistic regression	Female: OR = 0.5 (H=high), *p* < 0.01 (significant)	Not examined	Female: OR = 0.4 (middle) *p* = 0.01 (significant)
7	Ogba et al. (2025)	Nigeria	CS	Undergraduate students	1007	511, 50.8%	24.0 (2.18)	SSI	MSPSS	Hierarchical multiple regression	β = −0.48 *p* < 0.001 (significant)	β = −0.15 *p* < 0.01 (significant)	β = −0.24 *p* < 0.001 (significant)
8	Wei et al. (2024)	China	CS	College students	1897	1084 (57.1%)	19.92 (1.50)	HDSQ-SI	MSPSS	Regression	B = −0.009 *p* = 0.017 (significant)	B = −0.006 *p* = 0.108 (not significant)	Not examined

## Data Availability

The data presented in this study are available upon request from the corresponding author. The data are not publicly available due to privacy concerns.
